# The Influence of the Interaction between the rs1042713 ADRΒ2 Polymorphism and Dietary Insulin Indices on Cardiometabolic Risk Factors in Iranian Adults: Results from Fasa Adult Cohort Study (FACS)

**DOI:** 10.1016/j.cdnut.2026.107673

**Published:** 2026-03-17

**Authors:** Seyede Hamide Rajaie, Yaser Mansoori, Shiva Faghih, Sayyed Saeid Khayyatzadeh, Mohammad Mehdi Naghizadeh, Ali Kamel, Mojtaba Farjam, Reza Homayounfar, Hassan Mozaffari-Khosravi

**Affiliations:** 1 Department of Nutrition, School of Public Health, Shahid Sadoughi University of Medical Sciences, Yazd, Iran; 2 Department of Persian Medicine, Research Center for Traditional Medicine and History of Medicine, School of Medicine, Shiraz University of Medical Sciences, Shiraz, Iran; 3 Noncommunicable Diseases Research Center, Fasa University of Medical Sciences, Fasa, Iran; 4 Department of Medical Genetics, School of Medicine, Shiraz University of Medical Sciences, Shiraz, Iran; 5 Department of Community Nutrition, School of Nutrition and Food Sciences, Shiraz University of Medical Science, Shiraz, Iran; 6 Nutrition Research Center, Shiraz University of Medical Science, Shiraz, Iran; 7 Clinical Research Development Unit, Valiasr Hospital, Fasa University of Medical Sciences, Fasa, Iran; 8 National Nutrition and Food Technology Research Institute, Faculty of Nutrition Sciences and Food Technology, Shahid Beheshti University of Medical Sciences, Tehran, Iran

**Keywords:** ADRB2, rs1042713 SNP, SBP, HDL, cardiometabolic diseases, polymorphism

## Abstract

**Background:**

Cardiometabolic risk results from interactions between genetic and dietary factors. Variation in the adrenergic β-2 receptor gene (ADRB2 rs1042713) may influence responses to insulinogenic dietary patterns.

**Objectives:**

We aimed to assess whether the associations between dietary insulin indices (DIIs) and cardiometabolic risk factors differ by ADRB2 rs1042713 genotype.

**Methods:**

In this cross-sectional study involving 369 adults, the energy-adjusted DII and dietary insulin loads (DILs) were derived from a validated food frequency questionnaire. The rs1042713 polymorphism was genotyped using the polymerase chain reaction-restriction fragment length method. Gene-diet interactions were assessed using multivariable general linear models.

**Results:**

Significant interactions were observed between both DII and DIL and the rs1042713 genotype on systolic blood pressure (SBP) (*P*-interaction < 0.05). In tertile analyses, among A-allele carriers, SBP was 7.8–7.9 mm Hg in the highest versus lowest tertiles of both DIL and DII (95% CIs = 2.5, 13.2 mm Hg). In contrast, among GG homozygotes, SBP was slightly lower in the highest DII tertile compared with the lowest tertile. A modest interaction between DIL and genotype was observed for HDL cholesterol (*P* = 0.035), which was attenuated after adjustment (*P* = 0.056). Interactions with diastolic blood pressure were inconsistent.

**Conclusions:**

The associations between insulinogenic dietary patterns and SBP vary by ADRB2 rs1042713 genotype, with greater sensitivity among A-allele carriers and a more attenuated response among GG homozygotes. SBP emerged as the most consistent outcome, highlighting the need for confirmation in prospective studies.

## Introduction

Cardiometabolic diseases (CMDs) represent a major global health challenge [1]. Their incidence is increasing in both developed and developing countries, including Iran [2]. These complex and multifactorial diseases are influenced by interactions among genetic, environmental, and lifestyle factors [3].

The adrenergic β-2 receptor (ADRB2) polymorphisms are among the genetic variations potentially associated with various cardiovascular [4] and metabolic [[5], [6], [7], [8]] phenotypes. These polymorphisms have been shown to mediate physiological responses [9], including vasodilation [10], lipolysis [11,12], insulin secretion [13], insulin resistance [14], obesity [15,16], hypertension [17], and thermogenesis [18]. One of the most frequent ADRΒ2 polymorphisms, rs1042713 (Arg16Gly), is located at codon 16 of the ADRB2 gene on chromosome 5p31–32, and results in an arginine (Arg) to glycine (Gly) substitution [19].

The polymorphism rs1042713 has been linked to insulin resistance, a key factor in the development of CMDs [[20], [21], [22]]. Some studies show that individuals carrying the G allele of rs1042713 exhibit an increased risk of insulin resistance, suggesting a potential genetic predisposition to metabolic dysregulation, which may contribute to the pathogenesis of CMDs [8,20,23]. However, other studies have demonstrated that the A-allele of rs1042713 is associated with an increased risk of cardiometabolic risk factors [8,24].

Among environmental factors, dietary patterns that promote hyperinsulinemia—measured using DIIs such as the insulin index (II) and insulin load (IL)—have emerged as significant contributors to metabolic dysfunction [[25], [26], [27]].

Given that genetic factors, such as ADRB2 polymorphisms, and dietary components, including insulin indices, independently influence metabolic pathways involved in glucose regulation and lipid metabolism [[25], [26], [27]], investigating their interaction offers valuable insights into the variability of cardiometabolic risk among individuals.

The importance of gene-diet interactions lies in their potential to explain interindividual variability in metabolic responses to insulinogenic foods, thereby influencing susceptibility to CMDs. Recent studies investigating these interactions have demonstrated that genetic polymorphisms can modify the effects of dietary components on metabolic risk factors, whereas dietary factors may also modulate genetic influences [28]. For instance, the interaction between the ADRB2 rs1042713 polymorphism and specific fatty acid intake has been shown to affect glucose and insulin parameters, underscoring the gene’s role in modulating metabolic outcomes in response to diet [23].

Given the rising incidence of CMDs in both Iran and globally, elucidating the interaction between the ADRB2 rs1042713 polymorphism and DIIs may improve risk stratification, facilitate the development of personalized nutritional interventions, and ultimately contribute to effective preventive and therapeutic strategies aimed at reducing cardiometabolic risk among Iranian adults and broader populations [29]. However, few studies [23,30,31] have investigated the interactions between nutritional factors and the ADRB2 rs1042713 polymorphism. Therefore, we aimed to evaluate the effects of the interaction between the ADRB2 rs1042713 polymorphism and DIIs on cardiometabolic risk factors in Iranian adults.

## Methods

### Study population

The Fasa city branch of the Prospective Epidemiological Research Studies in IRAN (PERSIAN) cohort study provided the data for this cross-sectional investigation. At baseline, 10,138 adults, aged 35 to 70 y, living in the Sheshdeh region of Fasa (Sheshdeh town and 24 neighboring villages) participated in this cohort study based on a previously published protocol [32,33].

Of the 10,138 participants at baseline, 6356 were selected based on the following criteria: First, participants who under- or over-reported their calorie intake (<800 kcal/d or >4200 kcal/d; *n* = 1278), or had missing data for dietary intake (*n* = 20), or outcomes of interest (*n* = 15) were excluded. Additionally, because of potential dietary changes, participants who were pregnant, lactating, or who had a history of disorders such as diabetes, kidney failure, or hypertension were excluded (*n* = 2429). Furthermore, because they might underreport their nutritional consumption, participants with a BMI of ≥40 were disqualified (*n* = 40).

According to the study by Mollahosseini et al. [34], the mean and SD of triglycerides (TG)—which exhibited the greatest variability among cardiovascular risk factors—were 142 ± 104.8, with the smallest difference between tertiles being 11 units. Therefore, the minimum sample size needed to detect a difference of <11 units between tertiles with 95% CI was 349 samples.

$$n=\frac{s^{2}}{d^{2}}Z^{2}=\frac{104.8^{2}}{11^{2}}1.96^{2}=349$$

d = Mean difference
s = Standard deviation difference
Z1-α/2 = 1.96

A final, randomly selected sample of 369 participants was included.

### Dietary assessment

A validated, block-format, 125-item semi-quantitative food frequency questionnaire (FFQ) was used in face-to-face interviews with trained dieticians to determine the individual’s usual food intake over the course of the preceding year [35]. Participants were asked throughout the interview to indicate their frequency of food consumption per day, week, or month, based on household measurements. Then, weights (in grams) of the various portions were calculated. The USDA’s nutrient database, adapted for Iranian foods, was used to estimate the energy and nutrient content of the foods [36,37].

### DII and DIL

The food insulin index (FII) is quantified as the total insulin AUC measured over a 2-h period following the consumption of a 1000-kilojoule portion of the test food, normalized by the AUC obtained after ingestion of an equivalent energy portion of a reference food. Insulin index values for certain food items were obtained from previous studies [27,[38], [39], [40]], whereas indexes for other foods, including specific Iranian food items, were estimated by referencing the FII of analogous foods with similar energy, carbohydrate, protein, fat, and fiber compositions. For 3 particular items—coffee, tea, and salt—the FII was presumed to be zero, given their negligible content of energy, carbohydrates, proteins, and fats. The details are provided in Supplementary Table 1 [26].

The DIL for each participant was determined by aggregating the insulin load values of individual foods (FIL). The FIL for each food item was computed using the following formula [38]:

FIL = insulin index of the specific food × energy content per gram of the food × daily consumption amount in grams.

Subsequently, the DII for each participant was derived by normalizing the DIL with respect to the total daily energy intake [27]. Finally, both indices were adjusted for energy intake using a regression method to obtain energy-adjusted DIL and DII values.

### Anthropometric and blood pressure measurements

Weight and height were measured with the individual wearing light indoor clothing, to the nearest 0.1 kg and 0.5 cm, respectively, on a digital scale. Body weight (kg) divided by the square of the height (m^2^) was used to compute BMI. Waist circumference (WC) was measured at a point midway between the iliac crest and the lowest rib without applying pressure to the body, while the participant was exhaling [41]. The waist to height ratio (WHtR) was calculated by dividing the WC by height. Body composition [fat mass (kg and percent) and fat-free mass (kg) of the whole body] was measured using a Bioelectrical Impedance Analysis device (Tanita BC-418, Tanita Corp; Supplementary Table 2).

Participants were instructed to rest for 10 min before their blood pressure was measured using a standard mercury sphygmomanometer. Two blood pressure readings were taken from the right arm at 15-min intervals, and then the mean of these 2 measurements were reported as blood pressure value.

### Biochemical assays

Blood samples were obtained from participants following a 12- to 14-h overnight fasting period. The samples were collected, centrifuged, and divided into aliquots, which were labeled and stored at −70°C. In addition to the aliquoted samples, a portion of the blood was used to measure fasting blood glucose (FBG), TG, and HDL cholesterol (HDL-C) concentrations. All biochemical analyses were performed using an AutoAnalyzer system (Selectra E, Vitalab, Holliston; Supplementary Table 2) with Pars Azmoon assay kits

### Genotyping

The rs1042713 single nucleotide polymorphism (SNP) on chromosome 5p31–32 was genotyped using the polymerase chain reaction-restriction fragment length polymorphism (PCR-RFLP) method. Its major allele is G, whereas its minor allele is A. The DNG-plus extraction kit (Cinnagen; Supplementary Table 2) was used to extract DNA from whole blood in accordance with the instructions provided by the manufacturer.

The following PCR primers were used: forward 5'-GCCTTCTTGCTGGCACCCCAT-3'; reverse 5'-CAGACGCTCGAACTTGGCCATG-3'. PCR reactions were conducted using a DNA thermocycler in a final volume of 29.5 μL [containing 3 μL extracted DNA, 1.5 μL primers, 10 μL distilled water, and 15 μL Taq DNA Polymerase Master Mix (Ampliqon, Denmark); Supplementary Table 2]. The DNA thermocycler was set as follows: 45 cycles at 95°C, 60°C, and 72°C (each step lasting 30 s), after DNA templates were denatured at 95 C for 1 min. The size of the PCR product was 168 bp [42].

The NcoI restriction enzyme (Thermo Fisher Scientific; Supplementary Table 2) was used to digest amplified DNA (8 μL) at 37°C overnight. NcoI cuts 22 bp from the 3'-end of both alleles, and produces the 146 bp fragment; it also cuts 18 bp from the 5'-end of the Gly-16 allele, and produces the 128 bp fragment [20]. The 2.5% agarose gels were used to electrophorese restriction digests, which were stained with Safe DNA Gel to make them visible under UV light.

**FIGURE 1:**
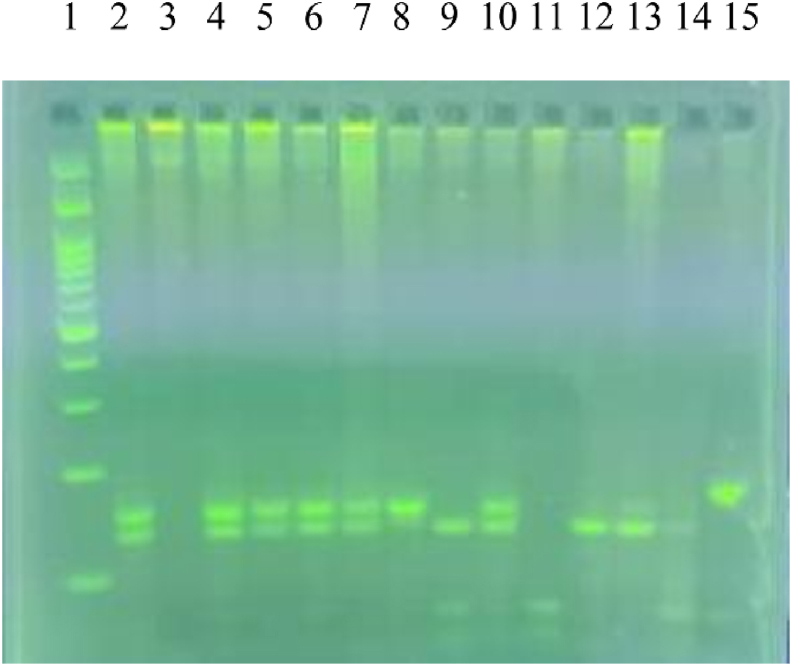
Agarose gel electrophoresis of PCR products showing the rs1042713 ADRB2 polymorphism. The ladder is in Lane 1; homozygotes for the glycine 16 allele (GG) are in Lanes 9, 12, and 13; homozygotes for the arginine 16 allele (AA) are in Lane 8; heterozygotes (AG) are in Lanes 2, 4, 5, 6, 7, and 10. Lane 15 contains the undigested PCR product.

Three genotypes were distinguished by attention to the length of the fragments that were produced during digestion: homozygous GG (128 bp); heterozygous GA (128 and 146 bp); and homozygous AA (146 bp). The 22 and 16 bp fragments were not visible (Figure 1).

### Assessment of other variables

Face-to-face interviews were conducted using a pretested questionnaire to record the following information: age (a continuous variable); socioeconomic status (SES) (continuous); sex (male/female); education (graduated from college or university compared with no college or university education); home ownership (owner compared with nonowner); marital status (married compared with single or divorced); and active smoking (currently smoking at least 1 cigarette per day). Physical activity (PA) was measured in metabolic equivalent hours per week (METs h/w) during the previous year [43]. SES was measured as the wealth score index [44] as reported elsewhere [26].

### Statistical analysis

Data were analyzed using IBM SPSS version 23 (IBM SPSS Corp.). *P* values < 0.05 were considered statistically significant. To assess potential selection bias, the baseline characteristics of participants included in the analysis were compared with those of all eligible participants prior to random sampling. These characteristics included age, sex, BMI, SBP, DII, and DIL (Supplementary Table 3).

The primary outcomes were the lipid profile [total cholesterol (TC), TG, HDL-C, and LDL cholesterol (LDL-C)], as well as systolic blood pressure (SBP) and diastolic blood pressure (DBP).

The normality of the distributions was assessed based on skewness and kurtosis [45]. Participants were classified into 3 categories according to energy-adjusted DIL and DII. Pearson’s Chi-square test was used to assess Hardy–Weinberg equilibrium for the SNP. The dominant model of ADRB2 rs1042713 was applied in the analyses. Independent samples t-tests, 1-way analysis of variance (ANOVA), or Chi-square tests were used to compare participant characteristics between genotypes or across categories of energy-adjusted DIL and DII, as appropriate.

In the primary analysis, both indices were also examined as continuous variables standardized to 1 SD increments. Genotype × diet interactions were assessed using multivariable linear regression models that included the main effects of genotype and DII or DIL (per SD), as well as their interaction term. Regression coefficients (β) are reported as the change in the outcome per SD increase in DII or DIL according to genotype, along with corresponding 95% CI. These continuous interaction models were employed to minimize information loss associated with categorization, enhance statistical efficiency, and facilitate comparability of effect estimates across outcomes.

In the secondary analysis, data were stratified into tertiles based on energy-adjusted DII and DIL scores. A general linear model was employed to examine the interaction between genotypes and adherence levels to energy-adjusted DIL and DII in both unadjusted and adjusted models. To evaluate differences in outcomes across categories of energy-adjusted DIL and DII within each genotype, analysis of variance (ANOVA) was conducted, followed by analysis of covariance (ANCOVA) to control for potential confounders. Sequential adjustment models were applied as follows: Model 1 adjusted for race, age, sex, physical activity (PA), education level, marital status, and smoking status; Model 2 additionally adjusted for energy-adjusted sodium intake to account for potential dietary confounding related to blood pressure; Model 3 further included centered BMI and centered PA, as well as interaction terms for genotype × BMI, genotype × PA, and genotype × sex, to explore potential effect modification.

For adjusted Model 3 (Supplementary Table 4), which includes various confounders, PA and BMI values were centered. Centering involves subtracting the mean value of a variable from each individual’s value. This process can reduce multicollinearity—especially between main effects and interaction terms—and improve the stability and interpretability of regression coefficients within the model [45,46].

Given the large number of statistical tests arising from the evaluation of genotype–diet interactions for 2 dietary insulin indices (DII and DIL) across multiple cardiometabolic outcomes and model specifications, control for multiple comparisons was applied. *P* values for genotype × diet interaction terms were adjusted using the Benjamini–Hochberg false discovery rate (FDR) procedure to limit the expected proportion of false positives [45]. Both nominal (unadjusted) *P* values and FDR-adjusted q-values are reported, with q < 0.05 considered statistically significant after correction for multiple testing.

## Results

**FIGURE 2:**
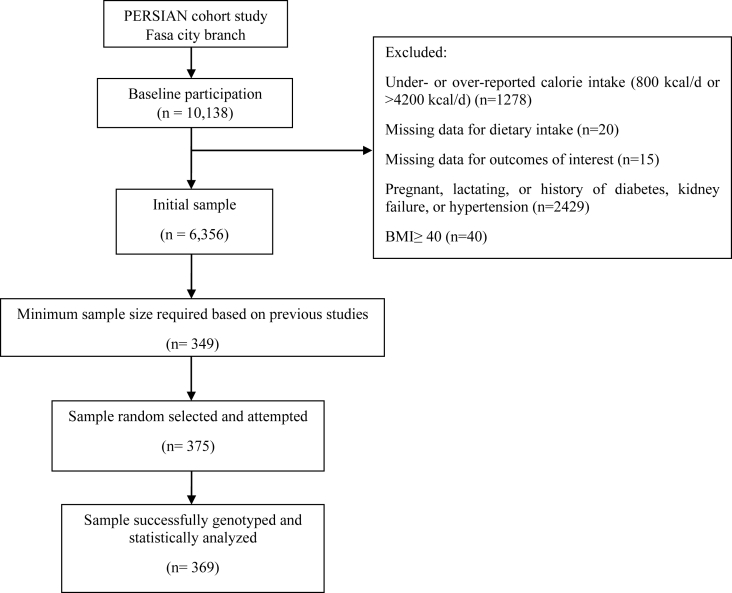
Flowchart illustrating the study population selection and exclusion criteria.

Of the 10,138 participants assessed at baseline, 6356 met the eligibility criteria. From these, 369 participants were randomly selected for the present analysis (Figure 2). Participants included in the present analysis were comparable to all eligible participants with respect to age, sex distribution, BMI, SBP, DII, and DIL (all *P* > 0.05; Supplementary Table 3), supporting the representativeness of the randomly selected subsample.

### Study population characteristics

A total of 369 individuals (mean age, 46.42 ± 8.57 y; mean BMI, 25.71 ± 4.51 kg/m^2^) were included in the analysis. The G allele was the most frequent variant (68.3%), and the minor allele frequency (MAF) was 31.7%. Genotyping quality control indicated a call rate >98%, 100% duplicate concordance (10% re-genotyping), and Hardy–Weinberg equilibrium (*P* = 0.828) (Table 1), supporting high data reliability (Supplementary Table 5).

**TABLE 1:** Genotype distribution and allele frequency of ADRB2 rs1042713.

Variable		*n* (%)	Hardy–Weinberg equilibrium	
			Chi-square statistic	*P* value
Genotype	AA	38 (10.3)	0.047	0.828
	AG	158 (42.8)		
	GG	173 (46.9)		
Allele	A	234 (31.7)	—	—
	G	504 (68.3)	—	—

Abbreviations: AA, homozygotes for the arginine; AG, heterozygotes; GG, homozygotes for the glycine.

We observed no statistically significant differences in the characteristics of the participants across the tertiles of the energy-adjusted DII and DIL (*P* > 0.05 for all) (Table 2). No significant associations were observed between adherence to the energy-adjusted DII and DIL and cardiometabolic risk factors, including biochemical and anthropometric indices (*P* > 0.05 for all) (Table 3).

**TABLE 2:** Characteristics of participants as a function of energy-adjusted insulin indices tertiles.

Variable	Tertiles of energy-adjusted DII score			*P* value	Tertiles of energy-adjusted DIL score			*P* value
	T1 (lowest)	T2	T3		T1 (lowest)	T1	T1	
*n*	123	123	123	—	123	123	123	—
Range	60.10≥	60.13- 65.30	≥65.37	—	138,585.96≥	138,595.69- 150,027.97	≥150,155.65	—
Qualitative variables^1^	*n* (%)	*n* (%)	*n* (%)		*n* (%)	*n* (%)	*n* (%)	
Sex (men)	60 (48.8)	65 (52.8)	62 (50.4)	0.814	64 (52.0)	61 (49.6)	62 (50.4)	0.927
Marital status (married)	111 (90.2)	113 (91.9)	112 (91.1)	0.905	112 (91.1)	111 (90.2)	113 (91.9)	0.905
Active smoker	38 (30.9)	39 (31.7)	35 (28.5)	0.846	40 (32.5)	39 (31.7)	33 (26.8)	0.576
Education (university graduate)	3 (2.4)	4 (3.3)	6 (4.9)	0.572	4 (3.3)	3 (2.4)	6 (4.9)	0.572
House ownership (owner)	109 (88.6)	115 (93.5)	106 (86.2)	0.164	108 (87.8)	116 (94.3)	106 (86.2)	0.090
Quantitave variables^2^	Mean ± SD	Mean ± SD	Mean ± SD		Mean ± SD	Mean ± SD	Mean ± SD	
Age (y)	45.22 ± 8.69	44.85 ± 8.58	46.20 ± 8.46	0.440	45.45 ± 8.79	44.77 ± 8.44	46.05 ± 8.49	0.507
Physical activity (MET-h/w)	44.41 ± 14.12	43.26 ± 12.64	42.39 ± 11.57	0.467	44.93 ± 14.21	42.14 ± 12.42	42.98 ± 11.60	0.216
Socioeconomic status (WSI)	0.11 ± 2.12	0.16 ± 2.31	0.32 ± 2.25	0.741	0.06 ± 2.17	0.19 ± 2.26	0.33 ± 2.26	0.640

Abbreviations: MET, metabolic equivalent; WSI, wealth score index.

1 P-values are derived from the chi-squared method.

2 P-values are derived from a 1-way analysis of variance (ANOVA) method for parametric variables, and the chi-squared method for nonparametric variables.

**TABLE 3:** Distribution of cardiometabolic risk factors according to tertiles of energy-adjusted DII and DIL.

Variable	Tertiles of and energy-adjusted DII score			*P* value^1^	*P* value^2^
	T1 (lowest)	T2	T3		
BMI (kg/m^2^)	25.50 ± 4.65	25.60 ± 4.28	26.02 ± 4.63	0.631	0.363
WC (cm)	92.25 ± 11.28	92.32 ± 11.04	93.81 ± 11.87	0.552	0.274
WHtR	0.57 ± 0.08	0.56 ± 0.07	0.58 ± 0.08	0.508	0.209
Percent body fat (%)	25.60 ± 10.18	27.62 ± 8.47	27.64 ± 9.80	0.315	0.477
Body fat mass (kg)	17.74 ± 8.78	19.96 ± 7.60	19.81 ± 9.27	0.208	0.478
Body fat-free mass (kg)	49.33 ± 8.33	51.06 ± 8.95	50.01 ± 9.59	0.497	0.639
SBP (mm Hg)	106.93 ± 15.07	108.29 ± 14.76	108.08 ± 15.64	0.750	0.852
DBP (mm Hg)	72.69 ± 11.39	74.08 ± 11.19	73.25 ± 10.63	0.613	0.641
FBS (mg/dL)	86.28 ± 9.01	88.25 ± 19.20	86.52 ± 10.98	0.474	0.318
TC (mg/dL)	181.26 ± 35.49	186.92 ± 38.53	187.29 ± 37.91	0.366	0.631
TG (mg/dL)	122.73 ± 71.71	134.38 ± 89.99	123.29 ± 58.05	0.384	0.909
LDL (mg/dL)	105.95 ± 28.04	108.77 ± 34.49	111.46 ± 31.98	0.394	0.515
HDL (mg/dL)	50.77 ± 16.10	51.28 ± 15.13	51.17 ± 14.26	0.962	0.684
Tertiles of and energy-adjusted DIL score					
BMI (kg/m^2^)	25.58 ± 4.54	25.38 ± 4.45	26.16 ± 4.57	0.372	0.771
WC (cm)	92.60 ± 11.25	91.74 ± 11.16	94.05 ± 11.74	0.270	0.691
WHtR	0.57 ± 0.08	0.56 ± 0.08	0.58 ± 0.08	0.328	0.348
Percent body fat (%)	25.70 ± 10.25	27.24 ± 8.58	27.88 ± 9.64	0.347	0.946
Body fat mass (kg)	18.02 ± 8.89	19.46 ± 7.72	20.00 ± 9.10	0.337	0.473
Body fat-free mass (kg)	49.77 ± 8.12	50.55 ± 9.20	50.04 ± 9.62	0.866	0.392
SBP (mm Hg)	107.05 ± 14.71	107.97 ± 15.14	108.28 ± 15.63	0.803	0.760
DBP (mm Hg)	72.85 ± 11.17	73.96 ± 11.50	73.22 ± 10.55	0.725	0.589
FBS (mg/dL)	86.19 ± 8.97	88.41 ± 19.39	86.44 ± 10.65	0.387	0.362
TC (mg/dL)	182.48 ± 34.99	186.01± 39.87	187.00 ± 37.13	0.609	0.363
TG (mg/dL)	127.71 ± 73.55	128.60 ± 88.63	124.09 ± 58.49	0.881	0.429
LDL (mg/dL)	106.42 ± 28.55	108.37 ± 34.38	111.38 ± 31.68	0.465	0.458
HDL (mg/dL)	50.51 ± 16.22	51.91 ± 15.29	50.80 ± 13.91	0.746	0.915

Abbreviations: DII, dietary insulin index; DBP, diastolic blood pressure; DIL, dietary insulin load; FBS, fasting blood sugar; SBP, systolic blood pressure; TC, total cholesterol; TG, triglyceride; WC, waist circumference; WHtR, waist to height ratio.

Data are presented as mean ± SD.

1 Derived from a 1-way analysis of variance (ANOVA).

2 Adjusted for race, age, sex, physical activity, education level, marital and smoking status using analysis of covariance (ANCOVA).

The general characteristics of participants with ADRB2 rs1042713 genotypes (dominant model) are presented in Table 4. Those in the A carrier group were more likely to have a higher education level compared with those with the GG genotype (*P* = 0.021). However, we observed no statistically significant differences in sex, age, PA, socioeconomic status, marital status, home ownership, or smoking status among the ADRB2 rs1042713 genotypes.

**TABLE 4:** Characteristics of participants as a function of ADRB2 rs1042713 gene variants.

Variable	ADRB2 rs1042713 (dominant model)		*P* value
	AA+AG	GG	
Qualitative variables^1^	*n* (%)	*n* (%)	
Sex (men)	102 (52.0)	85 (49.1)	0.577
Marital status (married)	179 (91.3)	157 (90.8)	0.847
Active smoker	64 (32.7)	48 (27.7)	0.306
Education (university graduate)	11 (5.6)	2 (1.2)	0.021
House ownership (owner)	171 (87.2)	159 (91.9)	0.146

Quantitative variables^2^	Mean ± SD	Mean ± SD	

Age (y)	45.4 ± 8.36	45.4 ± 8.83	0.960
Physical activity (MET-h/w)	43.13 ± 12.67	43.60 ± 13	0.720
Socioeconomic status (WSI)	−0.29 (−1.18–1.80)^3^	−0.64 (−1.45–0.99)	0.476^2^

Abbreviations: MET, metabolic equivalent; WSI; wealth score index.

1 P-values are derived from the chi-squared method.

2 P-values are derived from the independent samples *t* test method for parametric variables, and the chi-squared method for nonparametric variables.

3 Presented as median (IQR).

### The effect of interactions between ADRB2 rs1042713 genotypes and energy-adjusted DII and DIL adherence on cardiometabolic risk factors

#### Primary analysis: gene-diet interactions between rs1042713 and DIIs in continuous analyses

For each SD increase in the energy-adjusted DIL score, SBP was 0.20 mm Hg higher among A-allele carriers compared with those with the GG genotype (β = 0.202; 95% CI: 1.39, 7.43; *P* = 0.004; q = 0.026). A similar interaction was observed for the DII score (β = 0.21; 95% CI: 1.65, 7.66; *P* = 0.003; q = 0.026) (Table 5).

**TABLE 5:** Gene-diet interactions between rs1042713 and energy-adjusted dietary insulin indices in continuous analyses.

Variable	Dietary insulin indices	Crude model^1^		Model 1^2^		
		β (95% CI) per SD	p-interaction	β (95% CI) per SD	p-interaction	q-value (BH-FDR)^3^
**BMI**	DIL	0.02 (−0.81, 1.05)	0.800	0.03 (−0.61, 1.08)	0.586	1.000
	DII	0.06 (−1.39, 3.30)	0.058	0.003 (−0.57, 0.60)	0.960	1.000
**WC (cm)**	DIL	0.06 (−1.39, 3.30)	0.423	0.07 (−1.01, 3.63)	0.293	0.952
	DII	0.06 (−1.32, 3.36)	0.391	−0.021 (−1.075, 1.27)	0.753	1.000
**WHtR**	DIL	0.01 (−0.01, 0.02)	0.864	0.04 (−0.009, 0.02)	0.508	1.000
	DII	−0.02 (−0.01, 0.02)	0.796	−0.02 (−0.01, 0.008)	0.761	1.000
**Percent body fat (%)**	DIL	0.05 (−2.02, 3.31)	0.864	0.04 (−1.25, 2.28)	0.565	1.000
	DII	0.04 (−2.03, 3.23)	0.654	0.03 (−0.96, 1.63)	0.611	1.000
**Body fat mass (kg)**	DIL	0.03 (−2.02, 2.79)	0.751	0.03 (−1.64, 2.47)	0.691	1.000
	DII	0.05 (−1.78, 2.96)	0.622	0.04 (−1.15, 1.87)	0.638	1.000
**Body fat-free mass (kg)**	DIL	−0.08 (−3.49, 1.53)	0.442	−0.05 (−2.38, 1.06)	0.451	1.000
	DII	−0.05 (−3.16, 1.79)	0.586	0.07 (−0.63, 1.90)	0.325	1.000
**SBP (mm Hg)**	DIL	0.22 (1.79, 7.94)	0.002	0.20 (1.39, 7.43)	0.004	0.026
	DII	0.23 (1.91, −8.06)	0.002	0.21 (1.65, 7.66)	0.003	0.026
**DBP (mm Hg)**	DIL	0.12 (−0.29, 4.25)	0.087	0.11 (−0.54, 3.99)	0.012	0.052
	DII	0.13 (−0.14, 4.40)	0.066	−0.08 (−2.42, 0.72)	0.287	1.000
**FBS (mg/dL)**	DIL	−0.01 (−3.08, 2.60)	0.869	−0.04 (−3.50, 2.06)	0.610	1.000
	DII	0.004 (−2.76, 2.93)	0.954	0.02 (−1.61, 2.23)	0.752	1.000
**TC (mg/dL)**	DIL	0.06 (−4.25, 11.01)	0.384	0.05 (−4.56, 10.43)	0.441	1.000
	DII	0.06 (−4.15, 11.10)	0.371	0.03 (−4.19, 6.16)	0.709	1.000
**TG (mg/dL)**	DIL	−0.03 (−18.98, 11.68)	0.640	−0.04 (−19.58, 11.23)	0.594	1.000
	DII	−0.02 (−17.68, 12.99)	0.764	−0.01 (−11.62, 9.67)	0.857	1.000
**LDL (mg/dL)**	DIL	0.007 (−6.18, 6.78)	0.928	−0.004 (−6.51, 6.12)	0.951	1.000
	DII	0.01 (−6.00, 6.96)	0.884	0.08 (−1.94, 6.79)	0.275	1.000
**HDL (mg/dL)**	DIL	0.17 (−0.74, 6.89)	0.015	0.18 (0.94, 6.99)	0.002	0.026
	DII	0.16 (0.38, 6.54)	0.028	−0.08 (−3.34, 0.84)	0.243	1.000

Abbreviations: DII, dietary insulin index; DBP, diastolic blood pressure; DIL, dietary insulin load; FBS, fasting blood sugar; SBP, systolic blood pressure; TC, total cholesterol; TG, triglyceride; WC, waist circumference; WHtR, waist to height ratio.

1 Derived from the general linear model method (ANOVA).

2 Adjusted for race, age, sex, education level, marital and smoking status using the general linear model method (ANOVA).

3 False discovery rate (FDR) correction using the Benjamini–Hochberg procedure.

In addition, a significant interaction between rs1042713 and energy-adjusted DIL was detected for HDL. Each SD increase in energy-adjusted DIL was associated with a 0.18 mg/dL higher HDL concentration among A-allele carriers compared with those with the GG genotype (β = 0.18; 95% CI: 0.94, 6.99; *P* = 0.002; q = 0.026). The interaction between rs1042713 and energy-adjusted DII for HDL was not statistically significant (*P* = 0.243; q = 1.000) (Table 5).

For DBP, the interaction between rs1042713 and energy-adjusted DIL was significance (β = 0.11; 95% CI: −0.54, 3.99; *P* = 0.012; q = 0.052) only in crude model. No significant interactions were observed for other health outcomes (q > 0.50 for all) (Table 5).

#### Secondary analysis: gene-diet interactions between rs1042713 and tertiles of DII and load

Significant interactions were observed between adherence to the energy-adjusted DII and the rs1042713 SNP on mean DBP (*P*-interaction = 0.012), SBP (*P*-interaction = 0.001), and HDL concentrations (*P*-interaction = 0.002). These interactions remained statistically significant after adjusting for potential confounders (*P*-interaction < 0.05 for all). However, after FDR adjustment, interactions were observed only between adherence to the energy-adjusted DII and the rs1042713 SNP on mean SBP (*P*-interaction = 0.024) (Table 6).

**TABLE 6:** The effect of interactions between ADRB2 rs1042713 genotypes and energy-adjusted DII on cardiometabolic measures.

Variable	ADRB2 rs1042713 (dominant model)	Tertiles of energy-adjusted DII score		P value^1^	P value^2^	P- interaction^3^	P-interaction^4^	q-value (BH-FDR)^5^
		1 (lowest)	3					
**BMI**	**AA+AG**	25.41 ± 4.96	26.29 ± 4.53	0.539	0.992	0.800	0.470	0.870
	**GG**	25.60 ± 4.35	25.71 ± 4.76	0.981	0.635			
**WC (cm)**	**AA+AG**	92.04 ± 12.01	94.91 ± 12.18	0.348	0.449	0.628	0.305	0.724
	**GG**	92.47 ± 10.59	92.54 ± 11.48	0.946	0.883			
**WHtR**	**AA+AG**	0.57 ± 0.09	0.58 ± 0.09	0.473	0.546	0.796	0.547	0.870
	**GG**	0.57 ± 0.07	0.57 ± 0.08	0.921	0.946			
**Percent body fat (%)**	**AA+AG**	25.80 ± 10.35	28.93 ± 10.04	0.331	0.082	0.405	0.478	0.870
	**GG**	25.24 ± 10.05	25.07 ± 9.41	0.359	0.566			
**Body fat mass (kg)**	**AA+AG**	17.95 ± 9.01	20.70 ± 9.40	0.318	0.102	0.797	0.581	0.870
	**GG**	17.40 ± 8.52	18.74 ± 9.14	0.476	0.836			
**Body fat-free mass (kg)**	**AA+AG**	49.35 ± 8.39	49.03 ± 9.53	0.151	0.284	0.168	0.868	0.981
	**GG**	49.28 ± 8.38	51.20 ± 9.68	0.587	0.445			
**SBP (mm Hg)**	**AA+AG**	**102.60±13.03**	**110.40±16.98**	**0.014**	**0.040**	**0.002**	**0.001**	**0.024**
	**GG**	111.34±15.82	105.39±13.57	0.089	**0.047**			
**DBP (mm Hg)**	**AA+AG**	70.55 ± 11.31	74.76 ± 11.56	0.090	0.178	**0.027**	**0.012**	0.072
	**GG**	74.88 ± 11.14	71.51 ± 8.79	0.191	0.159			
**FBS (mg/dL)**	**AA+AG**	86.42 ± 8.79	87.08 ± 10.45	0.782	0.975	0.152	0.870	0.981
	**GG**	86.14 ± 9.30	85.87 ± 11.61	0.207	0.289			
**TC (mg/dL)**	**AA+AG**	174.90 ± 33.68	187.98 ± 39.24	0.109	0.117	0.196	0.118	0.708
	**GG**	174.90 ± 33.68	187.98 ± 39.24	0.501	0.706			
**TG (mg/dL)**	**AA+AG**	124.50 ± 69.85	121.89 ± 59.84	0.821	0.817	0.880	0.981	0.981
	**GG**	120.93 ± 69.85	121.89 ± 59.84	0.364	0.538			
**LDL (mg/dL)**	**AA+AG**	104.65 ± 26.79	111.36 ± 32.02	0.266	0.383	0.324	0.724	0.928
	**GG**	107.27 ± 29.41	111.57 ± 32.20	0.437	0.661			
**HDL (mg/dL)**	**AA+AG**	**45.35 ± 11.19**	**51.71 ± 15.02**	**0.029**	**0.043**	**0.004**	**0.002**	0.052
	**GG**	56.28 ± 18.40	50.55 ± 13.42	0.110	0.194			

Abbreviations: DII, dietary insulin index; DBP, diastolic blood pressure; FBS, fasting blood sugar; SBP, systolic blood pressure; TC, total cholesterol; TG, triglyceride; WC, waist circumference; WHtR, waist to height ratio.

1 Derived from a 1-way analysis of variance (ANOVA).

2 Adjusted for race, age, sex, education level, marital and smoking status using analysis of covariance (ANCOVA).

3 Derived from the general linear model method (ANOVA).

4 Adjusted for race, age, sex, education level, energy intake, marital and smoking status using the general linear model method (ANOVA).

5 False discovery rate (FDR) correction using the Benjamini–Hochberg procedure.

The mean SBP concentrations in the highest tertile of adherence to the energy-adjusted DII (high adherence) were significantly higher compared to those in the lowest tertile (low adherence), but only among A-allele carriers (*P* = 0.014 and *P* = 0.040 in crude and adjusted models, respectively). This association was not observed in individuals with the GG genotype (Table 6).

SBP was 7.8 mm Hg higher in the highest versus lowest DII tertile among A-allele carriers (95% CI: 2.5, 13.1). Although the point estimate indicates a moderate increase, bound of the CI suggests that effect could be small in some samples.

Adherence to the energy-adjusted DIL also showed a significant interaction with the rs1042713 SNP on SBP (*P*-interaction = 0.002) and HDL (*P*-interaction = 0.012) after adjusting for potential confounders. However, after FDR adjustment, the interaction remained significant only for mean SBP (*P*-interaction = 0.026). High adherence to the energy-adjusted DIL was associated with higher mean SBP in A-allele carriers (*P* = 0.014 and *P* = 0.035 in crude and adjusted models, respectively), whereas this relationship was not observed in individuals with the GG genotype (Table 7). The interactions between the rs1042713 SNP and the energy-adjusted DIL regarding other variables were not significant (*P*-interaction > 0.05).

**TABLE 7:** The effect of interactions between ADRB2 rs1042713 genotypes and energy-adjusted DIL on cardiometabolic measures.

Variable	ADRB2 rs1042713 (dominant model)	Tertiles of energy-adjusted DIL score		P value^1^	P value^2^	P-interaction^3^	P-interaction^4^	q-value (BH-FDR)^5^
		1 (lowest)	3					
**BMI**	**AA+AG**	25.54 ± 4.93	26.52 ± 4.42	0.293	0.327	0.406	0.739	1.213
	**GG**	25.63 ± 4.15	25.77 ± 4.74	0.936	0.903			
**WC (cm)**	**AA+AG**	92.39 ± 12.22	95.31 ± 11.98	0.216	0.305	0.298	0.653	1.212
	**GG**	92.81 ± 10.27	92.63±11.40	0.769	0.644			
**WHtR**	**AA+AG**	0.57 ± 0.09	0.58 ± 0.09	0.283	0.256	0.361	0.784	1.213
	**GG**	0.57 ± 0.07	0.57 ± 0.08	0.892	0.661			
**Percent body fat (%)**	**AA+AG**	25.88 ± 10.62	29.35 ± 9.68	0.210	0.312	0.386	0.670	1.212
	**GG**	25.42 ± 6.80	26.07 ± 9.41	0.411	0.882			
**Body fat mass (kg)**	**AA+AG**	18.23 ± 9.39	21.02 ± 9.05	0.336	0.423	0.362	0.851	1.213
	**GG**	17.67 ± 8.18	18.74 ± 9.14	0.599	0.981			
**Body fat-free mass (kg)**	**AA+AG**	49.68 ± 8.31	49.10 ± 9.59	0.292	0.537	0.887	0.512	1.131
	**GG**	49.92 ± 7.94	51.20 ± 9.68	0.513	0.438			
**SBP (mm Hg)**	**AA+AG**	**102.71 ± 13.26**	**110.63 ± 17.01**	**0.014**	**0.035**	0.826	**0.002**	**0.026**
	**GG**	111.46 ± 14.89	105.64 ± 13.58	0.101	0.060			
**DBP (mm Hg)**	**AA+AG**	70.92 ± 11.30	74.61 ± 11.81	0.166	0.269	0.710	0.065	0.282
	**GG**	74.81 ± 10.78	71.65 ± 8.78	0.231	0.198			
**FBS (mg/dL)**	**AA+AG**	86.20 ± 8.75	86.84 ± 9.84	0.923	0.945	0.296	0.348	1.131
	**GG**	86.18 ± 9.26	86.00 ± 11.55	0.234	0.205			
**TC (mg/dL)**	**AA+AG**	182.47 ± 34.98	186.00 ± 39.87	0.243	0.289	0.567	0.406	1.131
	**GG**	188.38 ± 36.20	187.00 ± 36.51	0.657	0.794			
**TG (mg/dL)**	**AA+AG**	131.57 ± 76.76	125.67 ± 58.07	0.865	0.841	0.856	0.701	1.213
	**GG**	123.79 ± 70.56	122.32 ± 59.40	0.627	0.688			
**LDL (mg/dL)**	**AA+AG**	104.94 ± 27.86	110.71 ± 31.44	0.380	0.439	0.467	0.784	1.213
	**GG**	107.93 ± 29.38	112.14 ± 32.21	0.608	0.733			
**HDL (mg/dL)**	**AA+AG**	45.41 ± 11.29	51.15 ± 14.49	0.035	0.056	0.717	**0.012**	0.078
	**GG**	55.69 ± 18.75	50.39 ± 13.35	0.177	0.222			

Abbreviations: BH-FDR, Benjamini–Hochberg false discovery rate; DBP, diastolic blood pressure; DIL, dietary insulin load; DBP, diastolic blood pressure; FBS, fasting blood sugar; SBP, systolic blood pressure; TC, total cholesterol; TG, triglyceride; WC, waist circumference; WHtR, waist to height ratio.

1 Derived from a 1-way analysis of variance (ANOVA).

2 Adjusted for race, age, sex, education level, marital and smoking status using analysis of covariance (ANCOVA).

3 Derived from the general linear model method (ANOVA).

4 Adjusted for race, age, sex, education level, energy intake, marital and smoking status using the general linear model method (ANOVA).

5 False discovery rate (FDR) correction using the Benjamini–Hochberg procedure.

Among A-allele carriers, SBP was 7.9 mm Hg higher in the highest compared with lowest DIL tertile (95% CI: 2.7, 13.2 mm Hg). Although the point estimate suggests a moderate increase, the lower bound of the CI indicates that the effect could be small in some samples.

### Effect-modification analyses

Adjustment for energy-adjusted sodium (Model 2) produced estimates virtually identical to those of Model 1 regarding the interaction between rs1042713 and dietary insulin indices (DII and DIL) on SBP and HDL, indicating that sodium intake did not confound the observed gene–diet interaction.

In the full interaction model (Model 3), which additionally included centered BMI, centered PA, and sex as effect-modifying variables, the interactions between rs1042713 and both DII and DIL on SBP remained significant (*P* = 0.003 for both DII and DIL), as did the interactions on HDL (*P* = 0.01 for both DII and DIL) (Supplementary Table 4).

However, none of the additional interaction terms between rs1042713 and cBMI, centered PA, or sex were statistically significant (*P* > 0.05) for either DII or DIL; therefore, these variables did not act as effect modifiers of the observed gene–diet association (Supplementary Table 4).

## Discussion

In this cross-sectional study, we examined whether the association between DIIs and cardiometabolic risk factors varied according to the ADRB2 rs1042713 polymorphism (minor allele frequency: 31.7%, comparable with British [47], Omani [48], and Caucasian [48] populations).

Primary analyses using continuous models revealed a statistically significant interaction between both the DII and DIL with ADRB2 rs1042713 on SBP, with each SD increase in energy-adjusted DII or DIL associated with approximately 0.20–0.21 mm Hg higher SBP among A-allele carriers compared with GG homozygotes. A similar interaction was observed between DIL and genotype for HDL, although the interaction with DII was not significant. Interactions with DBP were weaker and largely nonsignificant after adjustment, and no consistent interactions were detected for other cardiometabolic outcomes. Secondary analyses using tertiles of dietary insulin indices largely confirmed these patterns: among A-allele carriers, SBP was higher in the highest tertiles of DII or DIL compared with the lowest tertiles, whereas among GG homozygotes, SBP was slightly lower in the highest DII tertile than in the lowest tertile. Although point estimates indicated a moderate effect, CIs suggested variability in the magnitude of the association across populations. Collectively, these findings suggest that A-allele carriers may be more sensitive to dietary insulin exposure with respect to SBP regulation, whereas GG homozygotes may exhibit a comparatively more favorable or attenuated response. SBP emerged as the most consistent outcome, whereas interactions with HDL were modest and did not persist after correction for multiple testing.

To date, few studies have investigated the interactions between nutritional factors and the ADRB2 rs1042713 polymorphism. Previous studies [23,30,31], unlike the current one, have primarily focused on the interactions between this polymorphism and individual nutrients, rather than on its interactions with dietary patterns in relation to cardiometabolic outcomes.

Consistent with our findings of an attenuated response in GG homozygotes to lifestyle factors compared to A-allele carriers, a 4-mo trial evaluated the effects of 2 different hypoenergetic diets—low-fat (LF) and moderately high-protein (MHP)—on cholesterol changes in individuals carrying ADRB2 polymorphisms. In the MHP group, GG homozygotes exhibited smaller reductions in serum TC, LDL-C, and non–HDL-C compared with A-allele carriers. Conversely, in the LF group, GG homozygotes showed similar reductions in TC, LDL-C, and non–HDL-C compared with A-allele carriers [31]. Moreover, a cross-sectional study by Lagou et al. [15], involving 306 African-American and 315 European-American individuals, reported significant interactions between the ADRB2 rs1042713 polymorphism and vigorous PA on weight loss. GG homozygotes may benefit less from increased vigorous PA to reduce their weight [15]. However, a study involving 32 white normotensive individuals demonstrated that the ADRB2 rs1042713 polymorphism modulates the impact of dietary sodium restriction on cardiovascular function in healthy, normotensive individuals. Low-sodium diet (150 mmol/d) affected baseline myocardial function in GG homozygotes. Resting cardiac output decreased, stroke volume tended to decrease, SBP decreased, and systemic vascular resistance increased in the GG homozygotes. Conversely, these parameters remained largely unchanged in AA homozygotes after the low-sodium diet [30].

In addition, if we interpret our results to suggest that GG homozygotes may exhibit a comparatively more favorable response to lifestyle factors than A-allele carriers, consistent with our findings, it has been previously proposed that the G allele may be beneficial for cardiovascular health. A study involving 5888 individuals aged ≥65 y indicated a reduced risk of coronary events in G allele carriers compared to AA homozygotes [49]. Additionally, a prospective cohort study involving patients with acute coronary syndrome treated with β-blockers demonstrated a lower cumulative mortality rate over a 3-y period in GG homozygotes than in AA homozygotes [50]. Bao et al. [51] (2005) demonstrated that the haplotype containing the G allele conferred a protective effect against hypertension in European American adults <50 y. Taken together, the ADRB2 rs1042713 polymorphism exhibits significant pharmacogenetic relevance, along with notable effects on disease modulation and lifestyle factors.

A modest interaction was observed between DIL and ADRB2 genotypes on HDL concentrations, with A-allele carriers exhibiting slightly higher HDL in unadjusted models (*P* = 0.035) and borderline significance after adjustment (*P* = 0.056). Although this association did not remain significant after correction for multiple testing, it suggests that dietary insulin exposure may influence lipid metabolism in a genotype-dependent manner, warranting further investigation in larger or intervention-based studies.

The biological mechanisms underlying the observed gene–diet interactions are not yet fully understood; however, several plausible nutrigenetic and nutri-epigenetic pathways may help contextualize our findings. Diets characterized by a high DII or load induce repeated postprandial insulin responses, which can influence epigenetic regulation through DNA methylation, histone modification, and microRNA expression in metabolic and vascular tissues [52,53]. These insulin-related epigenetic effects may interact with functional variations in the ADRB2 gene to modify downstream adrenergic signaling. In this context, A-allele carriers may be more susceptible to insulin-mediated epigenetic modulation of β_2_-adrenergic and endothelial pathways, potentially resulting in attenuated vasodilatory responses and elevated SBP under insulinogenic dietary conditions. Conversely, GG homozygotes, who differ in receptor regulation and down-regulation kinetics, may exhibit a comparatively attenuated physiological response to diet-induced insulin exposure. Sympathetic nervous system activity and β-adrenergic signaling are also involved in lipid metabolism, particularly through the regulation of adipose tissue lipolysis and hepatic lipid flux, which in turn influence HDL remodeling via hepatic and lipoprotein lipase activity [[54], [55], [56], [57]]. Accordingly, genotype-dependent differences in adrenergic signaling may contribute to modest variations in HDL in response to insulinogenic diets, although these associations were weaker and did not consistently persist after correction for multiple testing. Overall, these mechanisms should be regarded as speculative and hypothesis-generating, requiring confirmation in experimental and longitudinal studies.

### Strengths

This study has several strengths. *1*) It investigates a biologically plausible gene-diet interaction between the ADRB2 rs1042713 polymorphism and dietary insulin indices (DII and DIL) in relation to cardiometabolic risk factors, an area that remains incompletely understood. By examining insulinogenic dietary patterns within the context of adrenergic signaling, the study extends prior nutrigenetic research beyond single nutrients. *2*) The analysis was conducted within a well-characterized, population-based cohort using standardized procedures for anthropometric measurements, blood pressure, biochemical assays, and genetic assessments. Genotyping quality was high, with call rates, duplicate concordance, and Hardy–Weinberg equilibrium evaluated and reported in the **Supplementary Materials**. *3*) Dietary insulin exposure was assessed using both the DII and DIL, which capture complementary aspects of diet-induced insulinemia. Importantly, these indices were modeled as continuous variables standardized per SD, and interaction effects were estimated using multivariable linear models reporting β coefficients and 95% CIs. This approach enhances statistical efficiency and interpretability compared to analyses based solely on categorical variables. *4*) The analyses further incorporated comprehensive covariate adjustments, including socio-demographic and lifestyle factors, energy-adjusted sodium intake, and an exploratory assessment of effect modification by sex, centered BMI, and centered PA. These modeling strategies enhance confidence that the observed interactions (particularly for SBP) are not attributable to major sources of confounding. *5*) Potential selection bias was addressed by comparing the characteristics of the analytic sample with those of all eligible participants prior to random subsampling, thereby supporting internal validity.

### Limitations

Several limitations warrant consideration. *1*) The cross-sectional design precludes any inference regarding temporality or causality; therefore, the findings should be interpreted as hypothesis-generating. *2*) Dietary intake was assessed using an FFQ, which is susceptible to recall bias and measurement error. The calculation of DIIs required imputing food insulin index values for certain items and assigning zero values to noncaloric foods (e.g., coffee, tea, and salt). Such nondifferential measurement errors may have attenuated true associations, particularly for lipid-related outcomes. *3*) Additionally, several exposures and covariates—including dietary intake and physical activity—were derived from self-reported questionnaires, which are inherently subjective despite validation. Objective or biological measures, such as dietary biomarkers, continuous glucose or insulin monitoring, or device-based PA assessments, could have complemented the questionnaire data and enhanced exposure characterization. *4*) The study population consisted of normotensive, nondiabetic adults, which resulted in limited variability in certain cardiometabolic outcomes. Although this reduces confounding from medication use or disease-related dietary modifications, it may limit the generalizability of the findings to higher-risk populations. *5*) Only one functional polymorphism in ADRB2 was examined. Although rs1042713 was selected based on prior biological and epidemiological evidence, including additional variants or negative-control polymorphisms would enhance genetic specificity. *6*) Although FDR correction was applied and emphasis was placed on estimation using confidence intervals, multiple interaction tests were conducted. Therefore, findings (particularly those with modest effect sizes) require confirmation in independent populations.

The present findings should be evaluated in well-designed cross-sectional studies or randomized controlled trials. Additionally, if future research demonstrates that A-allele carriers are more sensitive to the impact of lifestyle factors on cardiometabolic risk factors, it is recommended to examine whether the response of GG homozygotes to lifestyle factors is more favorable or more attenuated compared to that of A-allele carriers.

In conclusion, the association between DIIs and cardiometabolic risk factors, particularly SBP, appears to be modified by the ADRB2 rs1042713 polymorphism. Higher dietary insulin exposure, measured by the DII and DIL, was associated with higher SBP among A-allele carriers. In contrast, GG homozygotes exhibited a comparatively attenuated or more favorable SBP response, identifying SBP as the most consistent outcome in this relatively healthy population. Interactions with DBP and HDL cholesterol were weaker and did not consistently remain significant after correction for multiple testing, suggesting a more limited or context-dependent role for ADRB2 variation in these outcomes.

Overall, these findings support the hypothesis that genetic variation in adrenergic signaling may influence individual susceptibility to insulinogenic dietary patterns, particularly concerning blood pressure regulation. Although the effect sizes were modest and the cross-sectional design precludes causal inference, the results underscore the potential importance of genetic background in dietary risk stratification. Confirmation through prospective studies and randomized controlled trials is warranted to clarify underlying mechanisms and assess genotype-specific cardiovascular responses to dietary strategies targeting insulin exposure.

## Authors’ contributions

The authors’ responsibilities were as follows – MF managed data collection; HMK, RH and SF designed the research; HMK, RH and SSK managed the project and contributed to all steps; YM, AK, and SHR conducted genetic tests, MMN managed and SHR performed the statistical analysis and data interpretation; SHR wrote the first draft of the manuscript; SF, YM, MMN and SSK contributed to the preparation and finalization of the manuscript; HMK and RH had primary responsibility for the final content and all authors: read and approved the final manuscript.

## Data availability

The datasets used in this study are available from the corresponding author on reasonable request.

## Funding

The present article is taken from the PhD thesis prepared by Seyede Hamide Rajaie, and was financially supported by Shahid Sadoughi University of Medical Sciences, Yazd (grant number IR.SSU.SPH.REC.1399.168) and Fasa University of Medical Sciences (grant number IR.FUMS.REC.1399.145).

## Conflict of interest

The authors report no conflicts of interest.
