# Supplementary material for: The Influence of the Interaction between the rs1042713 ADRΒ2 Polymorphism and Dietary Insulin Indices on Cardiometabolic Risk Factors in Iranian Adults: Results from Fasa Adult Cohort Study (FACS)
Source: Curr Dev Nutr. 2026 Mar 17;10(5):107673. doi: 10.1016/j.cdnut.2026.107673 (PMC13141489; doi:10.1016/j.cdnut.2026.107673)
Supplement: multimedia component 1 [file mmc1.docx]

**The influence of the interaction between the rs1042713 ADRΒ2 polymorphism and dietary insulin indices on cardio-metabolic risk factors in Iranian adults: Results from Fasa Adult Cohort Study (FACS)**

**First author:** Seyede Hamide Rajaie

**Supplementary Table 1.** Food insulin index of food frequency questionnaire items, and references for their determination based on previous studies**.**

| FFQ Food Items | Reference Food Used for FII | FII per 1000 kJ (239 kcal) |
| --- | --- | --- |
| White bread (Lavash)^*^ | White bread^1^ | 5 ± 73 |
| Barbari bread^*^ | Grain bread^1^ | 4 ± 41 |
| Sangak bread^*^ | Grain bread^1^ | 4 ± 41 |
| Baguette bread^*^ | White bread^1^ | 5 ± 73 |
| “Tiri” local flatbread^*^ | White bread^1^ | 5 ± 73 |
| Diet bread^*^ | Grain bread^1^ | 4 ± 41 |
| Rice, cooked^*^ | White rice (cooked)^1^ | 9 ± 58 |
| Macaroni, cooked^*^ | Spiral pasta (cooked)^1^ | 4 ± 29 |
| Red meat^*^ | Beef^1^ | 12 ± 73 |
| Chicken meat^*^ | Chicken, pan-fried with skin^1^ | 4 ± 19 |
| Other poultry | Chicken, pan-fried with skin^2^ | 4 ± 19 |
| Tongue (beef/lamb) ^*^ | Lamb^2^ | 19 ± 41 |
| Fish^*^ | Fish^1^ | 13 ± 43 |
| Organ meat (beef/lamb) | Beef^1^ | 12 ± 37 |
| Chicken organs | Chicken, pan-fried with skin^1^ | 1 ± 4 |
| Canned tuna | Fish^1^ | 13 ± 43 |
| Brain (beef/lamb) | Lamb^2^ | 19 ± 41 |
| Egg^*^ | Egg^1^ | 4 ± 23 |
| Sausage^*^ | Beef sausage^1^ | 9 ± 7 |
| Hamburger^*^ | Ham, shaved (Coles)^1^ | 11 ± 19 |
| Kalleh Pacheh | Lamb^2^ | 19 ± 41 |
| Pizza^*^ | Pizza^1^ | 4 ± 47 |
| Milk^*^ | Milk^1^ | 3 ± 24 |
| Colostrum (Aghuz) | Yoghurt^2^ | 19 ± 46 |
| Yoghurt^*^ | Yoghurt^2^ | 19 ± 46 |
| Cheese^*^ | Cheese^1^ | 9 ± 33 |
| Doogh (yoghurt drink) ^*^ | Yoghurt^2^ | 19 ± 46 |
| Kashk (whey paste) | Yoghurt^2^ | 19 ± 46 |
| Lettuce | Broccoli^2^ | 8 ± 29 |
| Beans (legumes) ^*^ | Baked beans^1^ | 14 ± 88 |
| Cabbage^*^ | Broccoli^2^ | 8 ± 29 |
| Chickpeas^*^ | Steamed peas^1^ | 8 ± 37 |
| Lentils^*^ | Lentils^1^ | 9 ± 42 |
| Tomato | Broccoli^2^ | 8 ± 29 |
| Split peas^*^ | Steamed peas^2^ | 8 ± 37 |
| Soy^*^ | Tofu^1^ | 4 ± 21 |
| Broad beans | Mixed beans^2^ | 14 ± 34 |
| Cucumber | Broccoli^2^ | 8 ± 29 |
| Raw vegetables | Broccoli^2^ | 8 ± 29 |
| Local vegetables | Broccoli^1^ | 8 ± 29 |
| Tomato paste^*^ | Tomato pasta sauce^1^ | 8 ± 41 |
| Cooked vegetables (stewed) | Broccoli^2^ | 8 ± 29 |
| Pickled cucumber | Commercial coleslaw^1^ | 2 ± 20 |
| Squash & eggplant | Broccoli^2^ | 8 ± 29 |
| Celery | Steamed cauliflower^2^ | 9 ± 48 |
| Beet | Carrot^2^ | 7 ± 44 |
| Potato^*^ | Boiled potato^1^ | 8 ± 88 |
| Carrot^*^ | Carrot^2^ | 7 ± 44 |
| Garlic | Broccoli^2^ | 8 ± 29 |
| Green peas | Steamed peas^2^ | 8 ± 37 |
| Onion | Broccoli^2^ | 8 ± 29 |
| Corn^*^ | Popcorn^1^ | 7 ± 39 |
| Bell pepper | Broccoli^2^ | 8 ± 29 |
| Green beans | Steamed peas^2^ | 8 ± 37 |
| Mushroom | Broccoli^2^ | 8 ± 29 |
| Barley | Great Grains^1^ | 7 ± 57 |
| Pumpkin | Broccoli^2^ | 8 ± 29 |
| Wheat^*^ | All-Bran wheat flakes^1^ | 7 ± 55 |
| Hot pepper | Broccoli^2^ | 8 ± 29 |
| Cantaloupe^*^ | Melon^1^ | 15 ± 93 |
| Melon^*^ | Melon^1^ | 15 ± 93 |
| Watermelon | Melon^1^ | 15 ± 93 |
| Apricot | Raw peach^2^ | 18 ± 39 |
| Mango | Apple^1^ | 3 ± 43 |
| Cherry | Seedless raisins^1^ | 5 ± 31 |
| Peach^*^ | Raw peach^2^ | 18 ± 39 |
| Ghooreh | Seedless raisins^1^ | 5 ± 31 |
| Unripe almond (chaghala) | Seedless raisins^1^ | 5 ± 31 |
| Mulberries | Seedless raisins^1^ | 5 ± 31 |
| Strawberry | Orange^1^ | 2 ± 44 |
| Plum | Raw peach^1^ | 18 ± 39 |
| Fig | Seedless raisins^1^ | 5 ± 31 |
| Grapes^*^ | Black grapes^1^ | 4 ± 60 |
| Pear | Apple^1^ | 3 ± 43 |
| Apple^*^ | Apple^1^ | 3 ± 43 |
| Kiwi | Orange^1^ | 2 ± 44 |
| Citrus fruits^*^ | Orange^1^ | 2 ± 44 |
| Pomegranate | Orange^1^ | 2 ± 44 |
| Banana^*^ | Banana^1^ | 4 ± 59 |
| Persimmon | Orange^1^ | 2 ± 44 |
| Dates | Seedless raisins^1^ | 5 ± 31 |
| Natural fruit juice^*^ | Apple juice^1^ | 2 ± 72 |
| Dried fruits^*^ | Seedless raisins^1^ | 5 ± 31 |
| Raisins^*^ | Seedless raisins^1^ | 5 ± 31 |
| Fruit compote^*^ | Apple juice^1^ | 2 ± 47 |
| Lemon juice | Orange juice^1^ | 7 ± 55 |
| Margarine^*^ | Butter^1^ | 1 ± 2 |
| Butter^*^ | Butter^1^ | 1 ± 2 |
| Solid cooking fat^*^ | Butter^1^ | 1 ± 2 |
| Liquid oil | Olive oil^1^ | 1 ± 3 |
| Olive oil^*^ | Olive oil^1^ | 1 ± 3 |
| Olives | Walnut^1^ | 1 ± 5 |
| Mayonnaise | Cream^3^ | 8 ± 8 |
| Walnut^*^ | Walnut^1^ | 1 ± 5 |
| Peanuts^*^ | Salted roasted peanuts^1^ | 2 ± 15 |
| Other nuts (e.g., pistachio) | Salted roasted peanuts^1^ | 2 ± 15 |
| Seeds (sunflower, pumpkin) | Walnut^1^ | 1 ± 5 |
| Cream^*^ | Cream^2^ | 8 ± 8 |
| Candy^*^ | Glucose^1^ | 100 |
| Honey | Raspberry jam^1^ | 9 ± 62 |
| Jam^*^ | Raspberry jam^1^ | 9 ± 62 |
| Sugar^*^ | Glucose^1^ | 100 |
| Tea | — | 0 |
| Soft drink^*^ | Coca-Cola^1^ | 3 ± 44 |
| Malt beverage | Coca-Cola^1^ | 3 ± 44 |
| Coffee | — | 0 |
| Ice cream^*^ | Ice-cream^3^ | 9 ± 65 |
| Cookies (dry sweets) ^*^ | Cookie^3^ | 11 ± 67 |
| Cream-filled pastries | Apple pie^1^ | 4 ± 74 |
| Muscati dessert | Raisin bran cereal^1^ | 6 ± 96 |
| Gaz (Persian nougat) | Muesli bar^1^ | 4 ± 34 |
| Sekanjabin | Raspberry jam^1^ | 9 ± 62 |
| Chocolate | Mars bar^1^ | 11 ± 89 |
| Potato chips^*^ | Potato chips^3^ | 10 ± 44 |
| Puffed corn snack | Cornflakes^1^ | 6 ± 55 |
| Halva^*^ | Fat-free blueberry muffin^1^ | 6 ± 96 |
| Commercial fruit juice^*^ | Fruit punch^1^ | 10 ± 76 |
| Biscuit^*^ | Plain biscuit^2^ | 20 ± 48 |
| Pickles | Commercial coleslaw^1^ | 2 ± 20 |
| Pomegranate paste | Commercial coleslaw^1^ | 2 ± 20 |
| Salt | — | 0 |

**Abbreviations:** **FFQ**, food frequency questionnaire; **FII**, food Insulin Index.

FII values ​​are presented as mean ± standard error.

^*^ Obtained from previously published studies. The insulin index for three food items—tea, coffee, and salt—was set to zero because their energy, carbohydrate, protein, and fat contents are negligible. For other food items not included in the food lists of previous studies, FII of similar items was used, based on the correlation between their energy, fiber, carbohydrate, protein, and fat contents. FII was not determined for pasta and pizza cheese due to their very low average consumption.

^1^ Obtained from Bao et al. Study.

^2^Obtained from Bell et al. Study.

^3^Obtained from Holt et al. Study.

**Supplementary Table 2.** Equipment and materials used for biochemical and molecular analyses in the Fasa Persian Cohort

| **Product** | **Model** | **Manufacturer** | **Catalog number** | **Reagent grade** | **Lot information** |
| --- | --- | --- | --- | --- | --- |
| **Body Composition Analyzer** | BC‑418 MA | Tanita Corporation | Not provided | ____ | Serial No:15020586 |
| **AutoAnalyzer** | Selectra E **A**utoAnalyzer | ElitechGroup | Not provided | ____ | Not provided |
| **PCR Master Mixes** | Ampliqon PCR Master Mixes | Amoliqon | 2AE0201 | For molecular biology | 52003001250 |
| **NcoI Restriction Enzyme** | NcoI restriction endonuclease | **Thermo Fisher Scientific** | **ER0572** | For molecular biology | Not provided |
| **DNA Extraction Kit** | DNG-Plus DNA extraction kit | Cinnagen | EX600 | For molecular biology | 006001 |

**Supplementary Table 3.** Comparison of included participants and all eligible participants

| **Variable** | **Included (n = 369)** | **All eligible (n = 6,356)** | **P-value** |
| --- | --- | --- | --- |
| Age (years) | 46.06 ± 8.65 | 44.77 ± 8.47 | 0.59 |
| Sex (% men) | 187 (50.7) | 3178 (50) | 0.42 |
| BMI (kg/m²) | 24.13 ± 3.93 | 24.33 ± 4.52 | 0.32 |
| SBP (mmHg) | 107.77 ± 15.13 | 106.72 ± 15.11 | 0.20 |
| Energy-adjusted DII | 62.84 ± 6.89 | 62.38 ± 6.85 | 0.22 |
| Energy-adjusted DIL | 144386.57 ± 18118.68 | 144231.36 ± 14851.57 | 0.12 |

**Abbreviations:** **DII**, Dietary Insulin Index; **BMI**, Body mass index; **SBP**, systolic blood pressure.

Data are presented as mean ± standard deviation

**Supplementary Table 4.** Interaction effects between ADRB2 rs1042713 and Dietary Insulin Indices on primary outcomes across multivariable models

|  | **Primary outcomes** | **Models** | **Genotype×Diet P-value** | **Genotype× BMIc^1^**  **P-value** | **Genotype× PAc^2^**  **P-value** | **Genotype×sex**  **P-value** |
| --- | --- | --- | --- | --- | --- | --- |
| **DII** | **SBP (mmHg)** | Model 2^3^ | **0.003** | ____ | ____ | ____ |
|  |  | Model 3^4^ | **0.003** | 0.89 | 0.64 | 0.08 |
|  | **DBP (mmHg)** | Model 2 | 0.07 | ____ | ____ | ____ |
|  |  | Model 3 | 0.08 | 0.73 | 0.66 | 0.21 |
|  | **TC (mg/dl)** | Model 2 | 0.41 | ____ | ____ | ____ |
|  |  | Model 3 | 0.42 | 0.05 | 0.13 | 0.23 |
|  | **TG (mg/dl)** | Model 2 | 0.69 | ____ | ____ | ____ |
|  |  | Model 3 | 0.72 | 0.77 | 0.90 | 0.84 |
|  | **LDL (mg/dl)** | Model 2 | 0.76 | ____ | ____ | ____ |
|  |  | Model 3 | 0.73 | 0.05 | 0.25 | 0.31 |
|  | **HDL (mg/dl)** | Model 2 | **0.01** | ____ | ____ | ____ |
|  |  | Model 3 | **0.01** | 0.30 | 0.15 | 0.54 |
| **DIL** | **SBP (mmHg)** | Model 2 | **0.003** | ____ | ____ | ____ |
|  |  | Model 3 | **0.003** | 0.89 | 0.64 | 0.08 |
|  | **DBP (mmHg)** | Model 2 | 0.07 | ____ | ____ | ____ |
|  |  | Model 3 | 0.08 | 0.73 | 0.66 | 0.21 |
|  | **TC (mg/dl)** | Model 2 | 0.41 | ____ | ____ | ____ |
|  |  | Model 3 | 0.42 | 0.05 | 0.13 | 0.23 |
|  | **TG (mg/dl)** | Model 2 | 0.70 | ____ | ____ | ____ |
|  |  | Model 3 | 0.72 | 0.77 | 0.90 | 0.84 |
|  | **LDL (mg/dl)** | Model 2 | 0.77 | ____ | ____ | ____ |
|  |  | Model 3 | 0.73 | 0.05 | 0.24 | 0.31 |
|  | **HDL (mg/dl)** | Model 2 | **0.02** | ____ | ____ | ____ |
|  |  | Model 3 | **0.01** | 0.30 | 0.15 | 0.54 |

**Abbreviations:** **DII**, Dietary Insulin Index; **DIL**, Dietary Insulin Load; **BMI**, Body mass index; **PA**, physical activity; **SBP**, systolic blood pressure; **DBP**, diastolic blood pressure; **TC**, total cholesterol ;**TG**, triglyceride ;**LDL**, Low-density lipoprotein; **HDL**, High-density lipoprotein.

^1^ Centered BMI (Centered means subtracting the mean value of a variable from each individual’s value. This process can reduce multicollinearity and improve the stability of coefficients.)

^2^ Centered physical activity

^3^ Model 1 covariates (race, age, sex, physical activity, education level, marital and smoking status) + energy adjusted sodium intake using the GLM method.

^4^ Model 1 covariates (race, age, sex, physical activity, education level, marital and smoking status) + BMIc +PAc + interaction terms (genotype× BMIc, genotype× PAc, and genotype×Sex) using the GLM method.

**Supplementary Table 5.** Genotyping quality control metrics for ADRB2 rs1042713

| Quality Control Metric | Description | Value |
| --- | --- | --- |
| Call rate (%) | Percentage of samples successfully genotyped for rs1042713 | >98% |
| Number of samples genotyped | Total samples included after quality control | 369 |
| Missing genotype rate | Proportion of samples without a successful genotype call | <2% |
| Duplicate samples (%) | Proportion of samples randomly selected for duplicate genotyping | 10% |
| Duplicate concordance (%) | Concordance rate between original and duplicate genotypes | 100% |
| Genotyping method | PCR–RFLP assay using NcoI restriction digest | ___ |

**Abbreviations:** PCR-RFLP, Polymerase chain reaction-restriction fragment length polymorphism
